# Wide-range IR spectra of diarylethene derivatives and their simulation using the density functional theory

**DOI:** 10.1038/s41598-022-20264-x

**Published:** 2022-10-07

**Authors:** Arkadiusz Jarota, Daria Drwal, Jakub Pięta, Ewa Pastorczak

**Affiliations:** 1 Institute of Applied Radiation Chemistry, Lodz University of Technology, Wróblewskiego 15, 93-590 Łódź, Poland; 2 Institute of Physics, Lodz University of Technology, ul. Wólczańska 217/221, 93-005 Łódź, Poland

**Keywords:** Photochemistry, Physical chemistry, Computational chemistry

## Abstract

Diarylethenes (DAEs), promising photochromic molecular switches, undergo pericyclic reactions upon ultraviolet or visible light illumination. For this reason, most studies on DAEs employ UV–vis spectroscopies. However, also their infrared (IR) spectra are valuable, in particular, for understanding the vibrational dynamics which accompanies the relevant photoreactions. An accurate assignment of IR bands to molecular modes can be achieved through a comparison between experimental and computed theoretical spectra. Even though more sophisticated computational methods are available, the density functional theory (DFT) is usually employed for this task, because of its modest cost and versatility. Here, we have tested the ability of several DFT functionals to reproduce the wide-range, 400–3200 cm^−1^, IR spectra of open and closed isomers of four representative DAE molecules. We find that global and range-separated, corrected for anharmonicity by scaling factors, hybrid DFT functionals are able to reproduce the IR spectra of DAEs, however, instead of the popular B3LYP functional we propose the use of the dispersion-corrected PBE0 functional. The paper also proposes a semi-automatic method of band assignment.

## Introduction

Photochromic switches are compounds that undergo reversible structure rearrangements upon light illumination. The relevant achievable chemical forms feature distinct absorption spectra and other physicochemical parameters^1^. Thanks to these characteristics they have various potential applications in optical devices and spectroscopy^2–5^. One of the most promising groups of photoswitches are fluorinated diarylethenes (DAEs) which are thermally stable and have a relatively high fatigue resistance^6^. These compounds undergo a reversible pericyclic reaction. The open-ring forms of DAEs absorb only the ultraviolet (UV) light while the closed-ring isomers have optical absorption bands in both UV and visible spectral regions. One possible application of photoswitches are the alternative rewritable optical information storage devices^7,8^. In such devices, writing of information is performed by inducing a photochromic reaction of photochromic dye. For a non-destructive readout, the infrared (IR) light can be used since it does not initiate photoswitching^9,10^. For optimal employment of IR radiation, the spectrum of the light source should match the intense vibrational modes in the absorption spectra of open or closed isomers.

The dynamics of pertinent pericyclic reactions decides the potential applications of this class of photoswitches, hence its understanding becomes essential in materials design and applications. Modelling of dynamics of a photoreaction must involve an accurate computation of UV–vis spectra, but also of the molecular structure and of the IR frequencies. Since the electronic excitations are such essential features of diarylethenes, there is no shortage of theoretical and experimental studies on the UV–vis spectra of those systems. Even though to model the entire photochemical reaction of diarylethenes, multireference computational methods are needed^11,12^, the most popular approach for the spectrum simulations remains the time-dependent density functional theory (TD-DFT), due to its relative inexpensiveness and accuracy. Several exchange–correlation functionals, including CAM-B3LYP^13^, ωB97X-D^14^, PBE0^15,16^, M05-2X^17^, M06-2X^18^ were successfully applied to model the geometries, absorption spectra and 0–0 transition energies. While TD-DFT cannot correctly describe the potential energy surfaces near the conical intersections, where the photoreactions usually take place, it can still be applied to model the dynamics of the excited-state relaxation^19–22^. In fact, for larger diarylethene molecules, the dynamics simulation based on TD-DFT, in a moderately sized basis set, is often the most advanced, but still viable computational technique. For a correct simulation of the dynamics of a photoreaction, an accurate description of both the electronic and the vibrational spectrum is necessary. Since the accuracy of the electronic spectrum simulation by DFT has been well studied, we seek to show the performance of DFT for the IR spectra.

Here, we present experimental IR spectra of a few DAE derivatives in open-ring form and their comparison with IR spectra calculated using density functional theory (DFT) using several different functionals. Selecting the DFT functional for which the computed spectra fit best the experimental ones will significantly facilitate a correct interpretation of vibrational bands. This, in turn, will allow for better understanding of time-resolved vibrational experiments performed to determine the structural rearrangements accompanying the cyclization and cycloreversion in DAEs.

## Methodology

**Figure 1:**
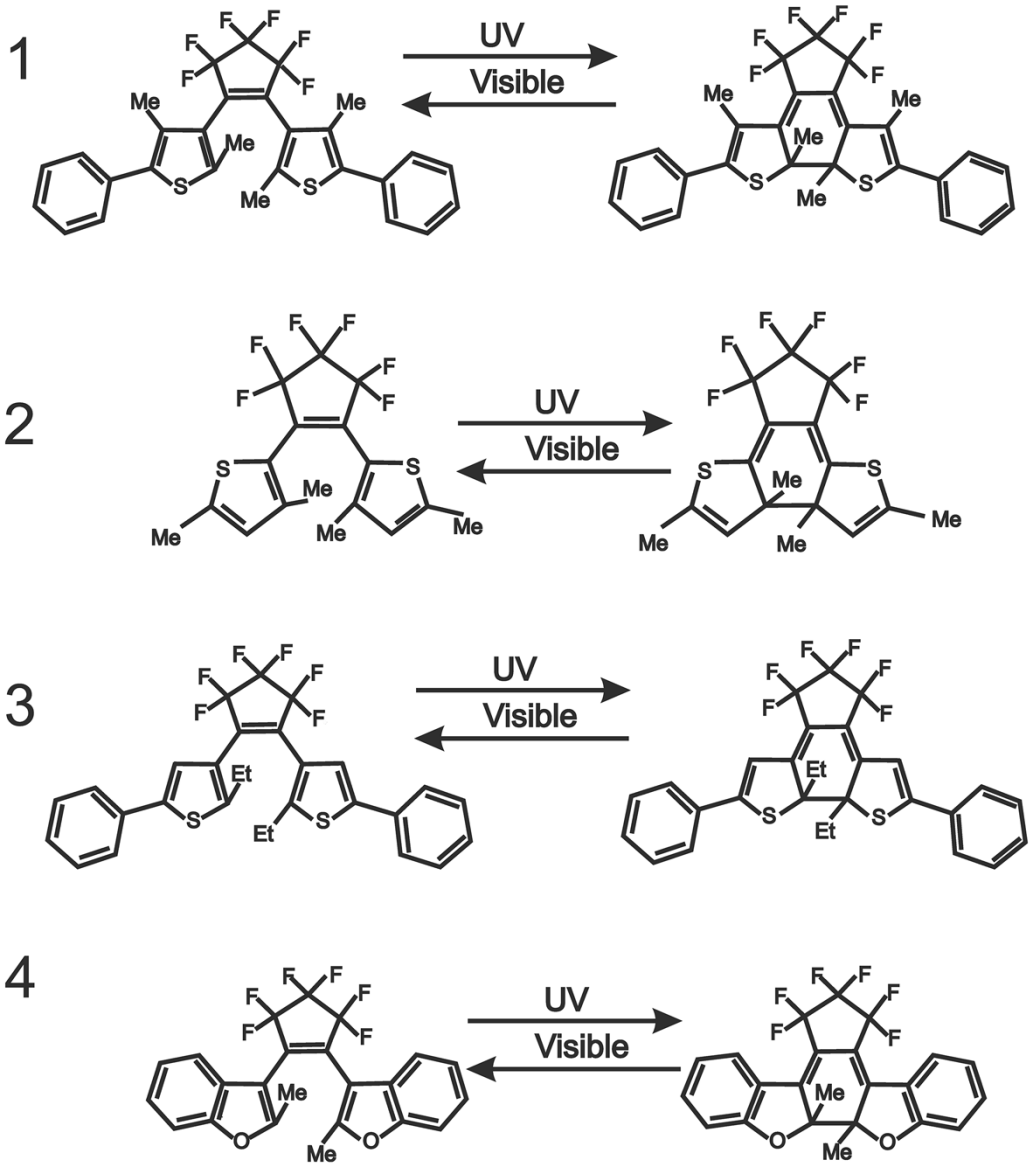
DAE derivatives and their photochemical reactions studied in this manuscript.

All diarylethenes have been purchased from Yamada Chemical Co. Ltd. (Kyoto). We have studied four DAE derivatives including 1,2-bis(2,4-dimethyl-5-phenyl-3-thienyl)perfluorocyclopentene (1), 1,2-bis(3,5-dimethylthiophen-2-yl)hexafluorocyclopentene (2), 1,2-bis(2-ethyl-5-phenyl-3-thienyl)perfluorocyclopentene (3), and 1,2-bis(2-methyl-1-benzofuran-3-yl)perfluorocyclopentene (4). Their chemical structures and photochemical reactions are shown in Fig. 1. The purities of compounds are the following as declared by the distributing company: (1)—99.8%, (2)—99.9%, (3)—99.8%, (4)—98%.

DAEs were dissolved in hexane to prepare the solutions of open-ring isomers. These solutions were irradiated for approximately two hours to perform the ring closure reaction using the output from femtosecond laser set-up (300 nm, ~ 1 µJ, ~ 150 fs) which is described elsewhere^22^. After the irradiation, the solutions contained both open and closed ring isomers that were subsequently separated using thin layer chromatography (TLC). All separations were performed on the PTLC (preparative TLC) silica gel 60 glass plates with fluorescent indicator F_254_, 1 mm, purchased from Sigma-Aldrich. Solvents used for separation and extraction of the products were purchased from Avantor Performance Materials Poland SA and used as received. The details of the separation procedure on TLC are included in Supplementary Information (SI). After the TLC, the solvent was let to evaporate, and the remaining powder was re-dissolved in CCl_4_. The CCl_4_ has been selected because its IR spectrum contains only a small number of high-intensity bands that can overwhelm the bands of the solute. The infrared spectra of DAEs were recorded using a Fourier-Transform IR (FTIR) spectrometer (Avatar 330, ThermoFisher Scientific) and demountable liquid cell (DLC, Harrick). The sample was placed between 2 mm thick KBr windows separated by 250 μm spacer.

The density functional theory computations were performed using the Gaussian 09D software^23^ and def2-SVP basis set^24^. The employed exchange–correlation functionals were chosen based on their popularity, cost (no double hybrids were included), stability of performance with respect to the grid and basis set, and previous proven performance for diarylethene UV–vis spectra and they include global hybrids B3LYP^25–28^, PBE0^29^, range-separated hybrids ωB97X-D^30^, LC-ωPBE^31–33^ and CAM-B3LYP^34^ and a local meta-GGA, M06L^35^. To assess the influence of the London dispersion interaction, functionals with and without the Grimme’s D3 dispersion correction^36^ were considered, except for ωB97X-D and M06L, which already account for dispersion without the correction. The open and closed structures of the studied molecules were optimized with the pertinent functionals and then each of the infrared spectra was calculated with the use of the same functional. To account for anharmonicity effects, the positions of IR peaks were adjusted by applying scaling factors^37^.

To compare the experimental (featuring broad vibrational bands) and the theoretical spectra (Lorentz-broadened with 4 cm^−1^ half-width at half maximum) with minimal bias, we have created a Python notebook^38,39^ script to serve as a semiautomatic band assignment and comparison tool for IR spectra. The algorithm of spectra comparison (whose code and input files are freely available on GitHub^40^) is as follows:

1. The experimental spectrum is read and normalized. The peaks are identified as implemented in the find_peaks Scipy^41,42^ procedure.
2. The theoretical spectrum is read and normalized. The peaks are identified as implemented in the find_peaks Scipy^42^ procedure.
3. Both experimental and simulated peaks are sorted in descending order by their heights.
4. *M* highest experimental peaks are then, one by one, attempted to be matched, to the nearest simulated peak which fulfills both the set proximity (*tol* = 40 cm^−1^ was chosen as the proximity threshold) and height criteria (value *htol* = 3 was chosen as permissible maximum ratio of theoretical to experimental peaks heights). The simulated peaks that have already been matched with an experimental peak are moved to the “Assigned” category, so they do not get matched to two different peaks.
5. If a match fulfilling the criteria cannot be found, the program prints that information and shows the closest matching theoretical peak.

In our analysis, approximately *M* = 20 of the most intense bands in the experimental spectrum in the range of 400–3500 cm^−1^ have been considered. Due to the large overlap of some of the peaks in the DAE spectra, a higher *M* would lead to a poorer certainty in the band assignment. However, if one only uses the provided script as a supportive tool for manual band assignment, it is entirely possible to choose a higher peak number. After determining the maxima locations of these bands and of the corresponding predicted frequencies from the theoretical spectrum using the above algorithm, the mean absolute errors (MAE) have been calculated according to Eq. (1) to estimate the accuracy of selected DFT functionals.

1 $$MAE=\frac{\sum \left|{\upsilon }_{predict}-{\upsilon }_{exp}\right|}{N}$$

where *N* is the number of matched peaks taken for analysis (*N* ≤ *M)* ${\upsilon }_{predict}$ is the (scaled) simulated peak frequency and ${\upsilon }_{exp}$ is a maximum of the experimental peak.

## Results and discussion

**Figure 2:**
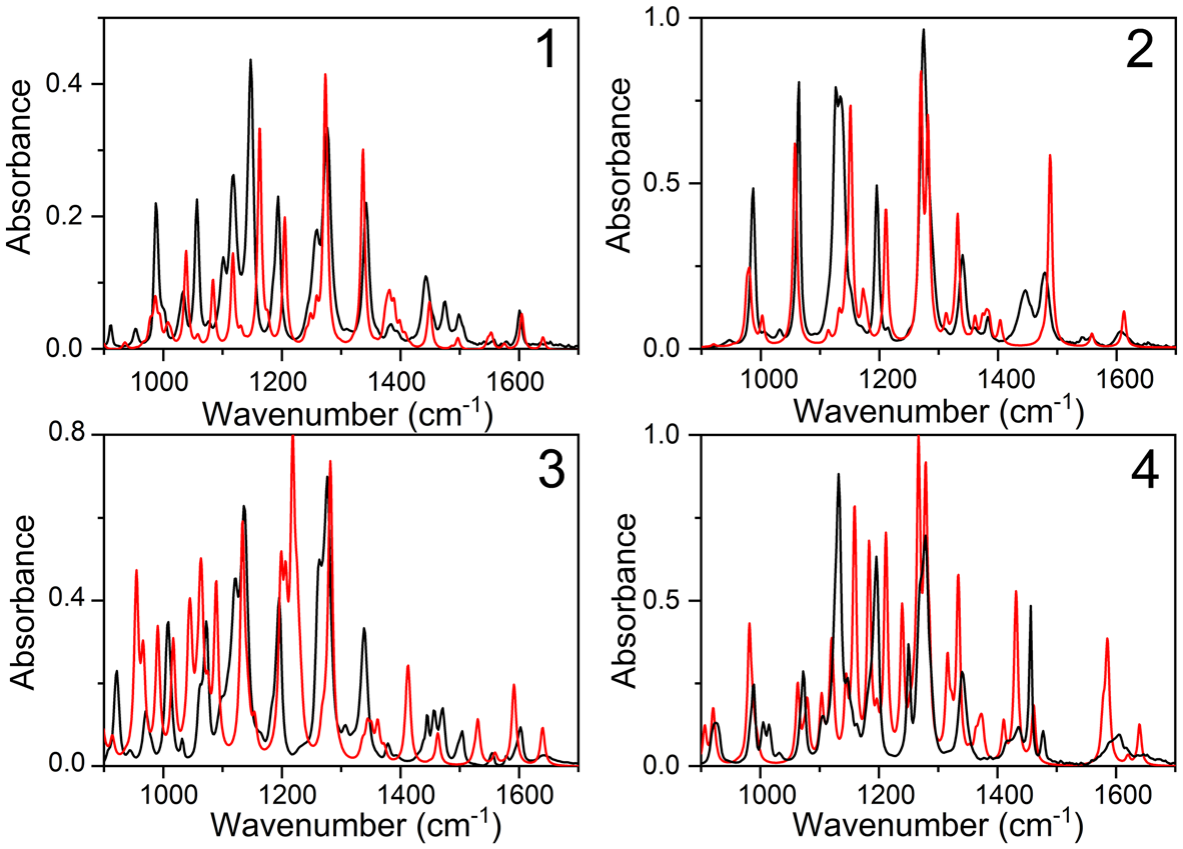
The experimental (black lines) and simulated using PBE0-D3 functional (red lines) IR spectra of open ring isomers of DAEs. The theoretical spectra were scaled using the following scaling factors: PBE0: 0.950, PBE0-D3: 0.950, B3LYP: 0.959, B3LYP-D3: 0.959, CAM-B3LYP: 0.951, CAM-B3LYP-D3: 0.951, M06L: 0.951, ωB97X-D: 0.950, LC-ωPBE: 0.9491, LC-ωPBE-D3: 0.9491.

**Figure 3:**
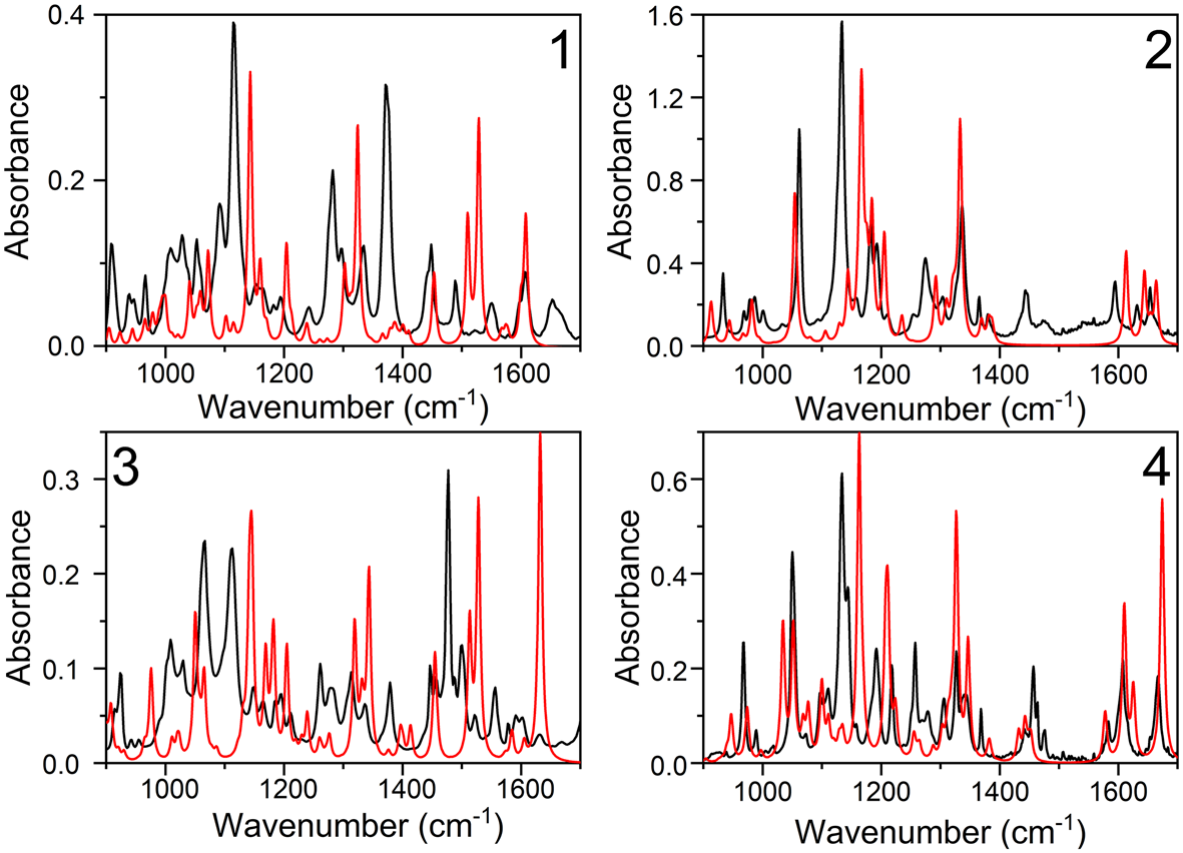
The experimental (black lines) and simulated using PBE0-D3 functional (red lines) IR spectra of closed ring isomers of DAEs. The theoretical spectra were scaled using the same scaling factors as in case of open ring isomer (see caption of Fig. 2).

**Figure 4:**
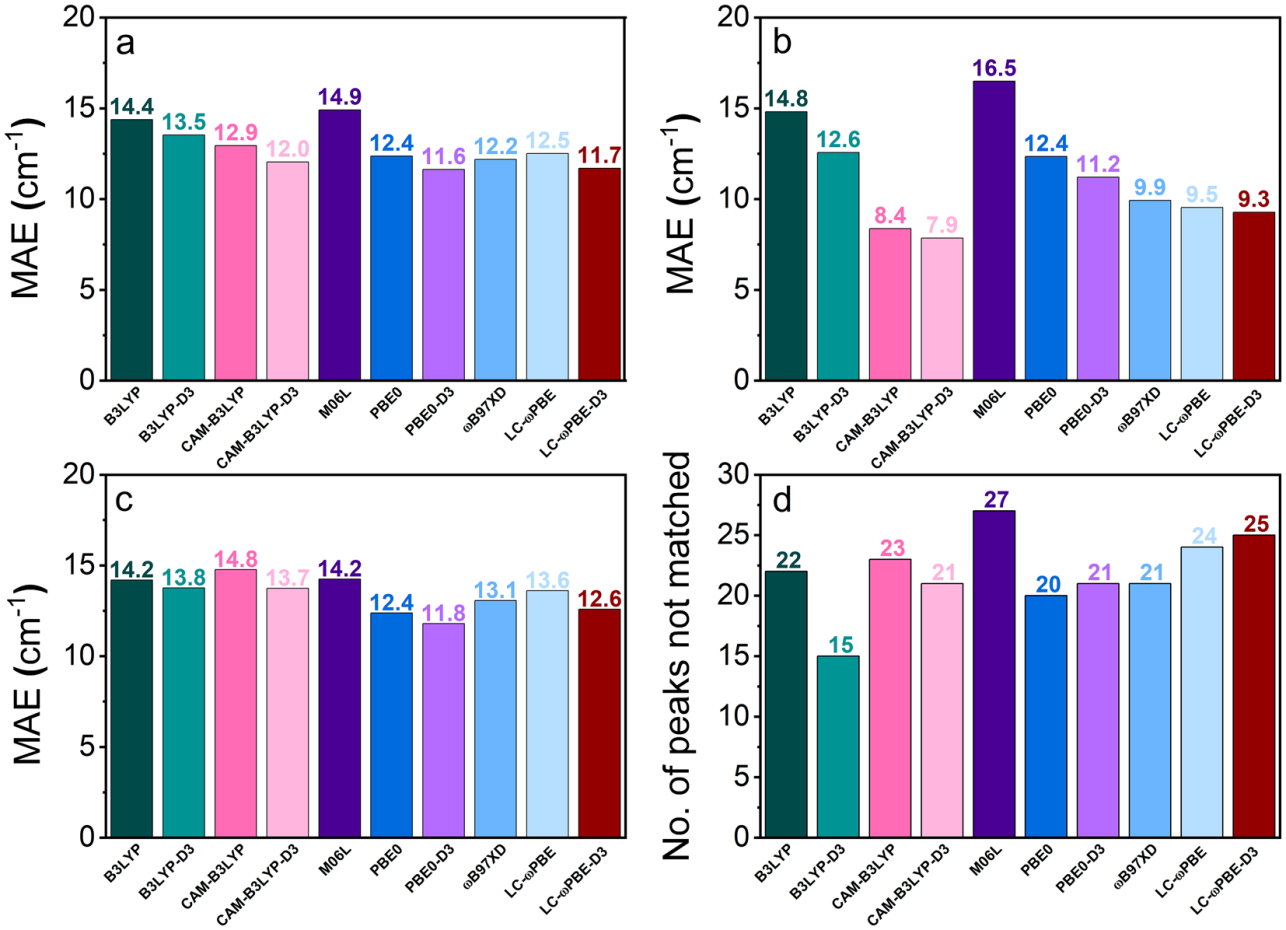
The MAEs of theoretical frequencies for various DFT functionals for (**a**) full-range spectra (400–3200 cm^−1^), (**b**) low wavenumbers (400–1000 cm^−1^), (**c**) high wavenumbers (over 1000 cm^−1^) and (**d**) the number of peaks that were not matched in experimental spectra.

The experimental and computed IR spectra of DAEs are presented in Fig. 2 (in open form) and Fig. 3 (in closed form). The results of MAE calculations for both open and closed isomers of DAEs for wavenumbers larger than 1000 cm^−1^ are gathered in Fig. 4b. This spectral range deserves the most attention since molecular vibrations involving carbon atoms reactive in photochromic reactions appear around 1500–1700 cm^−1^^44^. The low-frequency modes are also important because they are believed to facilitate the reaction by driving a DAE towards the photoproduct on the excited-state potential energy surface^45^. The relevant MAEs for the low-frequency spectral range, i.e. 400–1000 cm^−1^ are shown in Fig. 4c.

Clearly, another marker of the functional’s performance is the number of simulated peaks that could be matched with experimental ones with the adopted tolerance threshold. Hence, we show also the number of peaks that the functional “missed” out of the attempted 160 matches in Fig. 4d. This is only a supplementary criterion which qualifies the MAE value, i.e. when the value of MAE for given functional is minute, but the number of matched peaks is significantly smaller than that of the functional’s competitors, the functional’s performance cannot be considered excellent.

From Fig. 4a, where the MAEs of each functional, averaged across all molecules are plotted, it is clear that the global and range-separated hybrid functionals describe the IR spectra of diarylethenes with sufficient accuracy to identify the most important bands, even when a modest basis set is used. While the MAEs for the full range of IR spectrum for different functionals do not differ massively, the dispersion-corrected PBE0 functional is the best performer when it comes to the MAE, while B3LYP-D3 identifies most peaks (145 overall) with acceptable accuracy. The M06L functional produces both the largest error and identifies the fewest peaks (133). Looking at just the number of matched peaks, the functionals B3LYP, PBE0, PBE0-D3, CAM-B3LYP, CAM-B3LYP-D3 and ωB97X-D are on par with each other, having identified 138, 140, 139, 139, 137, 139 peaks, respectively, which means it is fair to compare their MAEs.

Larger differences between functionals can be spotted when looking at the MAEs for the lower-wavenumber part of the spectrum (Fig. 4b). The worst performer in that range is still the M06L functional, however the best turns out to be the dispersion-corrected CAM-B3LYP. In fact, the dispersion-corrected functionals are, in both spectral ranges, more robust than their uncorrected counterparts. While in the higher-frequency part of the spectrum the differences are not as pronounced, the PBE0-D3 functional achieves the best result, while uncorrected CAM-B3LYP—the poorest.

One could wonder whether, with such small differences between MAEs for each functional, and with several parameters that one needs to choose for the spectra comparison, those MAE differences are in fact meaningful. To that end, we have tested the performance of all the functionals for the DAE open-ring isomer (see Supplementary Information, Tables S2a and S2b) and the good performance of the PBE0-D3 functional, as well as the poor performance of M06L, can be observed across all parameter sets. Similarly, the superiority of the dispersion-corrected functionals is consistent for differently chosen *M*, *htol* and *tol* parameters.

Finally, the analysis of mean signed errors (MSE), see Table S3 in the Supplementary Information, shows that all of the functionals produce spectra shifted towards lower frequencies with respect to the experimental results.

Apart from finding the best functional, the other purpose for creating the software was assisting in the band assignment for the DAE derivatives. To that end, we show the selected vibrational modes in the Supplementary Information (Table S1). Naturally, the particularly interesting vibrations are the ones associated with the photoreaction, mainly those with a central ring (CR) C–C bond stretching component.

The closed isomers of molecules (2) and (3) have fairly intense IR bands associated CR stretching. Molecule (2) has a purely stretching CR mode at 851 cm^−1^ as well as composite, but involving the CR deformation bands at 1134, 1275, 1275, 1336 and 1595 cm^−1^, while molecule (3) has a range of vibrations involving the CR stretching in the range 1000–1200 cm^−1^ with a particularly intense peak at 1030 cm^−1^. On the other hand, molecule (1) has a low-intensity peak associated with a stretching mode at 922 cm^−1^ and a few others which also involve the CR stretching in the range 1100–1200 cm^−1^ as well as one around 1600 cm^−1^ which could not be precisely assigned due to the multitude of peaks in this region, however, neither of them is particularly intense. Even though the structure of molecule (4) differs significantly from the other molecules, the bands involving the CR deformation can be found in the same regions, i.e. at 1607 cm^−1^ as well as at 968 cm^−1^, and at 1192 cm^−1^ and at 1327 cm^−1^. The latter three bands have high intensities so their growth and disappearance can be used to track the relevant photoreactions.

## Conclusions

We have presented wide-range IR spectra of closed and open isomers of four different DAEs and we analyzed how accurately the most popular density functionals reproduce them. We find, that in fact, when the standard scaling factors are applied, the IR spectra are correctly reproduced with global hybrid and range-separated hybrid functionals, especially in the fingerprint and double bond (1500–2000 cm^−1^) regions. The only local functional tested, M06L, performs worst when it comes to MAE and the number of peaks accurately matched. Interestingly, the most popular functional in organic chemistry, B3LYP when not corrected for dispersion, turns out to be one of the poorer performers in the tested set and significantly shifts the spectra towards lower frequencies. As an alternative to B3LYP, for computationally expensive tasks, we propose the dispersion-corrected PBE0 functional, which has the same computational cost as B3LYP but is more reliable than the latter. Other possible choices include the range-separated hybrids, i.e. ωB97X-D and CAM-B3LYP functionals or the dispersion-corrected B3LYP which also performs quite well. Due to the presence of a solvent as well as the resolution of the experimental spectra, the differences in the functionals’ performances have to be considered very moderate, however, they are consistent across molecules and parameter sets. Each of those functionals is known to be capable of describing the non-covalent interactions, and their good performance in predicting the molecular structures and UV–vis of DAEs has been shown previously.

The IR spectra of DAEs are fairly dense with often indistinguishable bands located closely to each other. Therefore, regardless of the used method of simulation, some of the bands will not be possible to identify. However, for the purposes of tracking photoreactions, and checking for isomer contamination, any of the tested dispersion-corrected functionals, paired with either our script or an experienced researcher should be sufficient.

## Supplementary Information

Supplementary Information 1.

Supplementary Information 2.

## Data Availability

The datasets generated and/or analyzed during the current study are available in the GitHub repository: https://github.com/ewapastorczak/Wide-range-IR-spectra (a script to serve as a semiautomatic band assignment and comparison tool for IR spectra and input files) and as a Supplementary Information (all Gaussian output files).
