# Supplementary material for: Wide-range IR spectra of diarylethene derivatives and their simulation using the density functional theory
Source: Sci Rep. 2022 Oct 7;12:16834. doi: 10.1038/s41598-022-20264-x (PMC9546887; doi:10.1038/s41598-022-20264-x)
Supplement: Supplementary file 2 — Supplementary Information 2. [file 41598_2022_20264_MOESM2_ESM.docx]

Supplementary Information for article

“Wide-range IR spectra of diarylethene derivatives and their simulation using the Density Functional Theory”

by Arkadiusz Jarota^a^, Daria Drwal^b^, Jakub Pięta^a^, Ewa Pastorczak^b^

1. The details of TLC separation procedure

Preparative TLC plates were prepared as follows: starting line was set at 1.5 cm from the bottom of the plate. Appropriate mixture containing product of cyclization was applied on the starting line as a hexane solution with a Pasteur pipette. Subsequently, each plate was developed repeatedly in a rectangular glass chamber, with hexane as a mobile phase, 6 times. After development the plate was dried and appropriate spots were scratched off with a metal spatula. Silica gel containing desired product was transferred to flask equipped with a magnetic stirring bar and a proper volume of a 20% methanol in DCM solution was added. After 20 minutes of vigorous mixing the solution was transferred through a syringe filter (PTFE, 20 µm, 25 mm Ø) to a flask, solvents were removed under reduced pressure and the sample was left to dry.

1. The IR spectra of DAEs in 400-900 cm^-1^ and 2700-3200 cm^-1^ spectral ranges.





Fig. S1 The experimental (black lines) and simulated using PBE0-D3 functional (red lines) IR spectra of closed ring isomers of DAEs.





Fig. S2 The experimental (black lines) and simulated using PBE0-D3 functional (red lines) IR spectra of closed ring isomers of DAEs.





Fig.S3 The experimental (black lines) and simulated using PBE0-D3 functional (red lines) IR spectra of open ring isomers of DAEs.





Fig.S4 The experimental (black lines) and simulated using PBE0-D3 functional (red lines) IR spectra of open ring isomers of DAEs.

| 1. The assignments of IR bands | | |
| --- | --- | --- |
|  | | |
| Table S1. The assignments of selected IR bands | | |
| Molecule (2) open isomer | | |
| Scaled Vibrational frequency (cm^-1^) | Vibrational frequency  and its graphical representation | Assignment |
| 545 (555) | 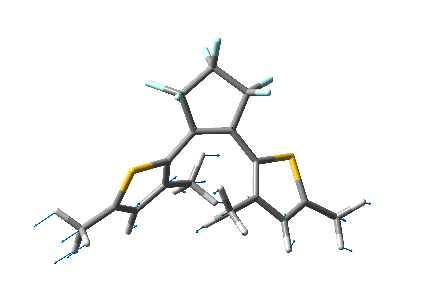 | ν(CC)_Thio_+ δ(FCF)_Per_ |
| 980 (987) | 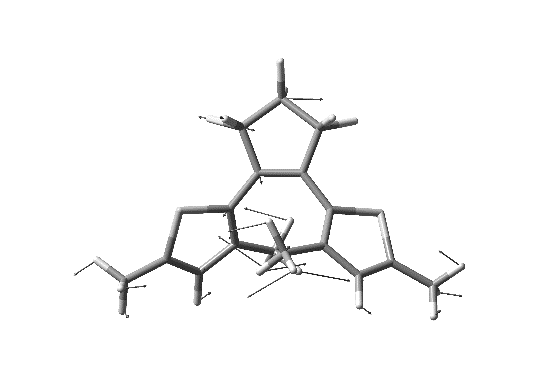 | τ(CH_3_)_Thio_+ δ(CCC)_Per_ |
| 1056 (1065) | 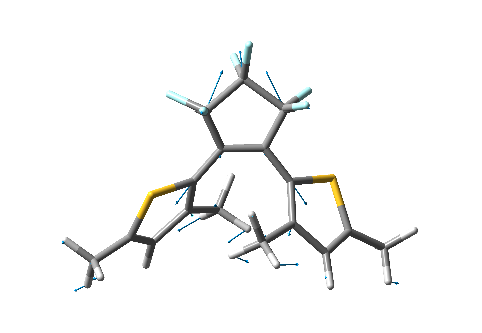 | δ(CCC)_Thio_+ ν(C-C)_Per_ |
| 1151 (1126) | 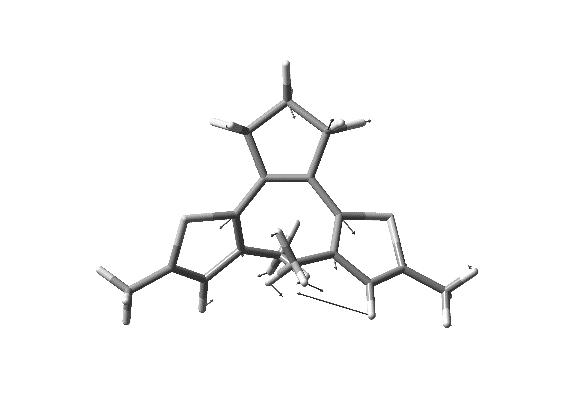 | γ_ring(Per)_+ ν(CC)_Thio_+ρ(CH)_Thio_+ω(CH_3_)_Thio_ |
| 1210 (1196) | 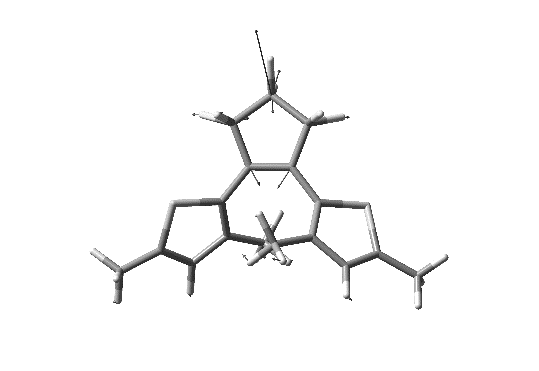 | γ_ring(Per)_+ ω(CH_3_)_Thio_ |
| 1270 (1275) | 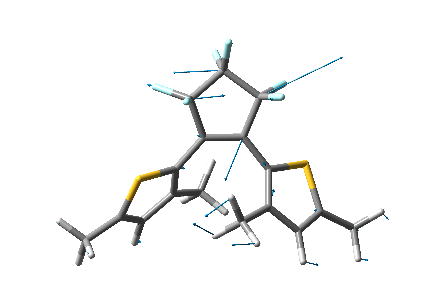 | ν(CC)_Per_+γ(CH3)_Thio_+ γ(CH_3_)_Thio_ |
| 1488 (1479) | 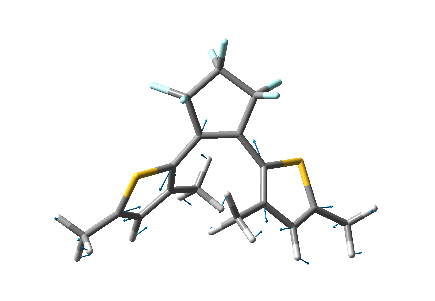 | ν(CC)_Thio_ |
| ν: stretching, δ: in-plane bending vibration, γ: bending out-of-plane vibration (breathing), τ: out-of-plane bending (twisting), ρ: in-plane bending (rocking) , ω: out-of-plane bending (wagging) Thio: thiophene, Per: perfluorocyclopentene | | |

| Molecule (2) closed isomer | | |
| --- | --- | --- |
| Vibrational frequency (cm^-1^) | Vibrational frequency  and its graphical representation | Assignment |
| 833 (851) | 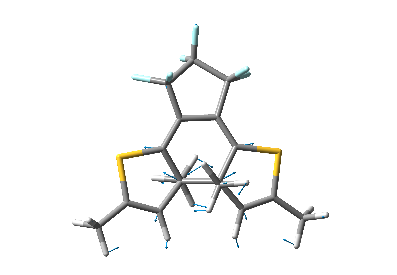 | ν(CC)_CR_ |
| 1054 (1062) | 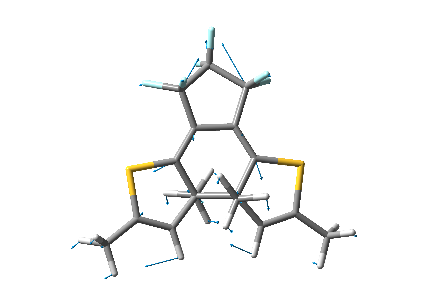 | δ(CCC)_Per_+ δ(CCH)_Thio_ |
| 1166 (1134) | 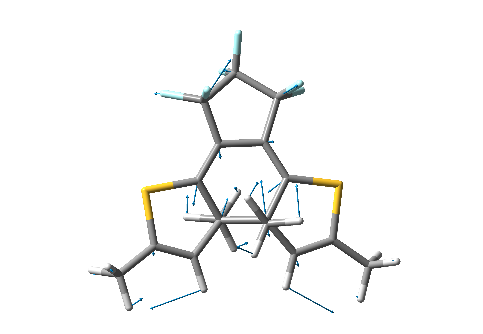 | γ_ring(Per)_+ ν(CC)_CR_+ δ(CCH)_Thio_ |
| 1183 (1182) | 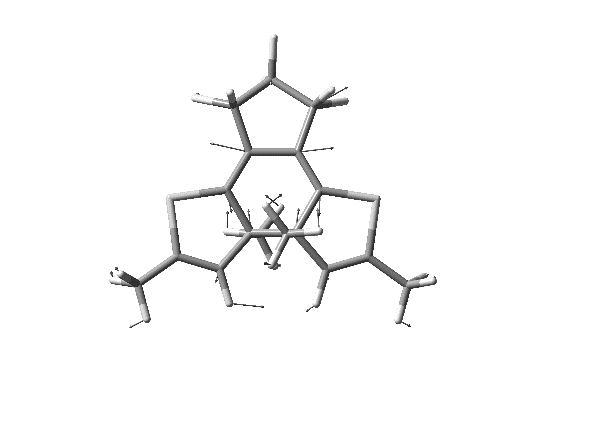 | ν(CC)_Per_+ ω(CH_3_)_Thio_+ τ(CH_3_)_Thio_ |
| 1207 (1193) | 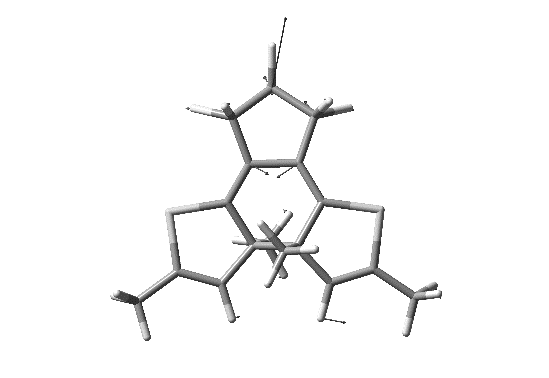 | δ(CCC)_Per_ |
| 1290 (1275) | 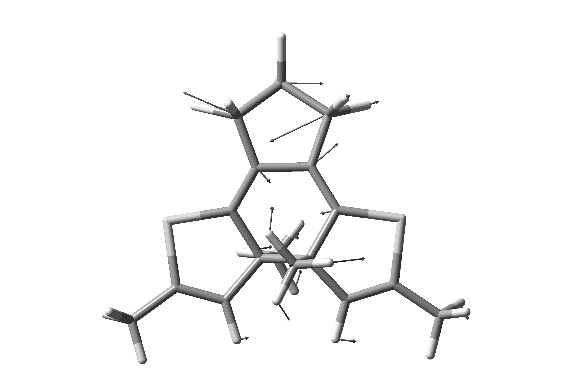 | ν(CC)_Per_+ δ(CCC)_CR_ |
| 1332 (1336) | 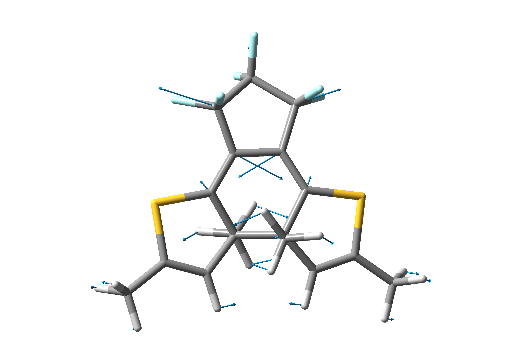 | ν(CC)_CR_+ ν(CC)_Per_ |
| 1613 (1595) | 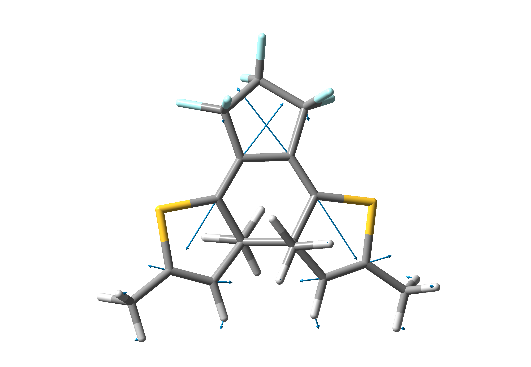 | ν(CC)_CR_+ ν(CC)_Pe_r+ ν(CC)_Tio_ |
| ν: stretching vibration, δ: in-plane bending vibration, γ: bending out-of-plane vibration (breathing), ω: out-of-plane bending (wagging), τ: out-of-plane bending (twisting) Thio: thiophene , Per: perfluorocyclopentene, CR: central ring | | |

| Molecule (1) open isomer | | |
| --- | --- | --- |
| Vibrational frequency (cm^-1^) | Vibrational frequency  and its graphical representation | Assignment |
| 1038 (1057) | 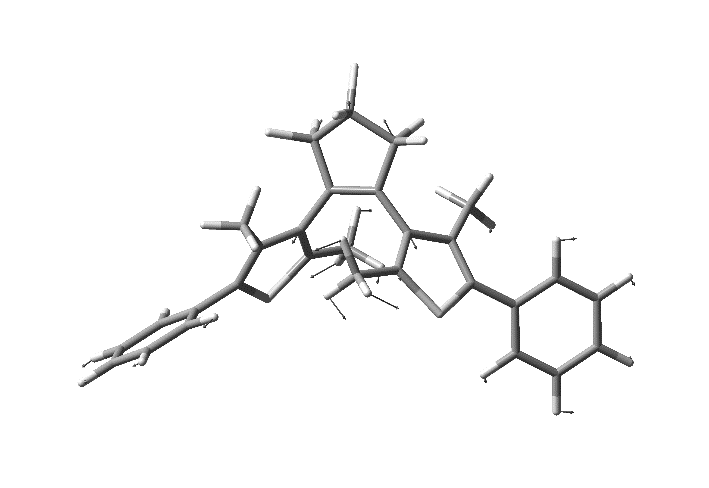 | ν(CC)_Per_+ ν(CC)_Thio_+ τ(CH)_Thio_+ρ(CH)_Ph_ |
| 1117 (1119) | 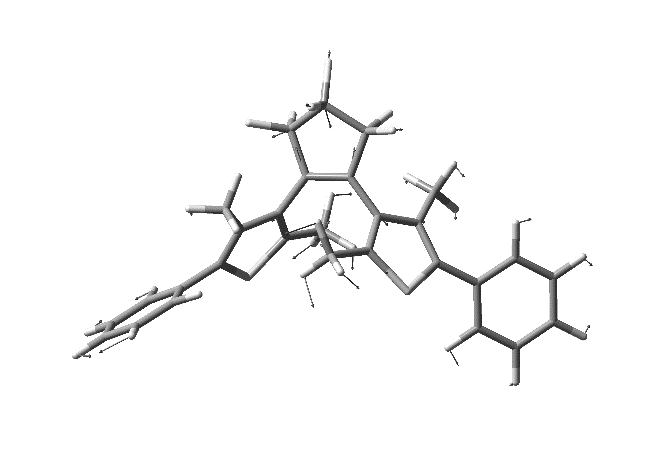 | δ(CCC)_Per_+ ρ(CH)_Ph_ |
| 1162 (1148) | 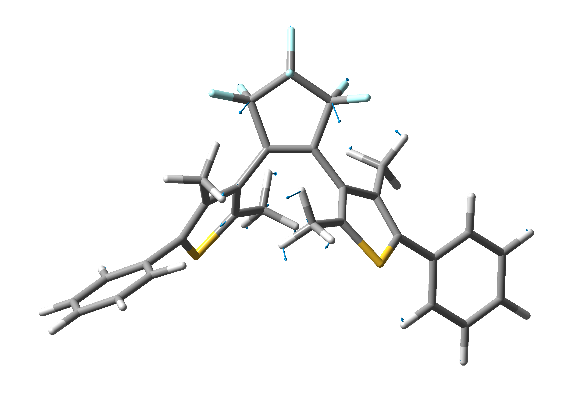 | γ_ring(Per)_+γ(CH_3_)_Tio_ |
| 1205 (1194) | 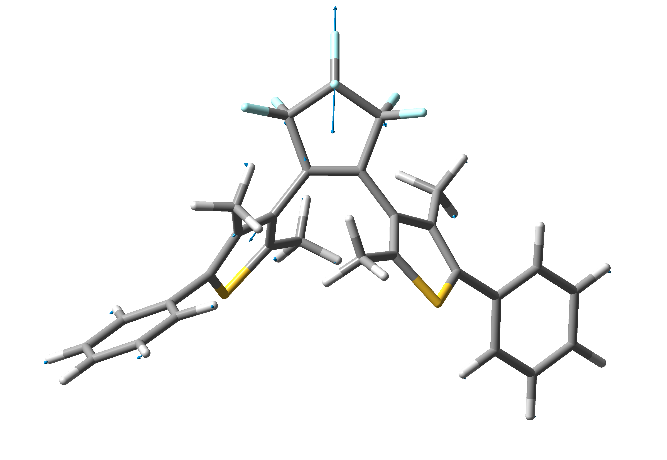 | γ(CCF)_Per_ |
| 1273 (1277) | 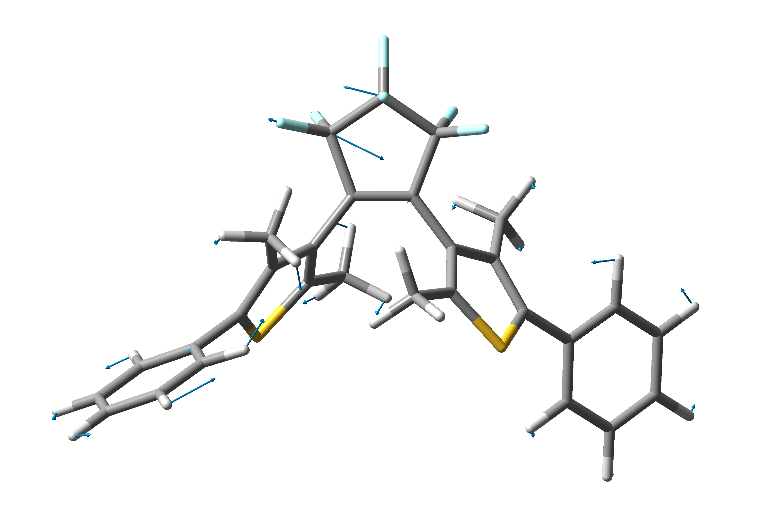 | ν(CC)_Per_+ρ(CH)_Ph_ |
| 1336 (1342) | 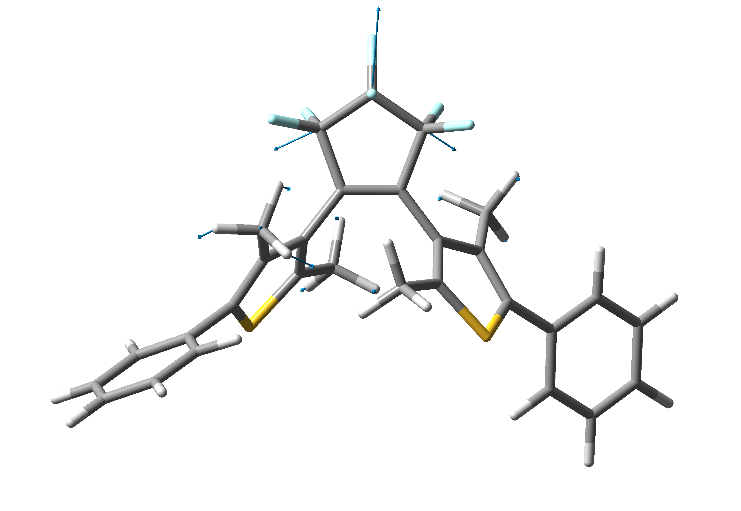 | ν(CC)_Per_+ γ(CH_3_)_Thio_ |
| ν: stretching, δ: in-plane bending vibration (scissoring), γ: bending out-of-plane vibration (breathing), ω: out-of-plane bending (wagging), ρ: in-plane bending (rocking) Thio: thiophene, Per: perfluorocyclopentene, Ph: phenyl | | |

| Molecule (3) open isomer | | |
| --- | --- | --- |
| Vibrational frequency (cm^-1^) | Vibrational frequency  and its graphical representation | Assignment |
| 992 (1009) | 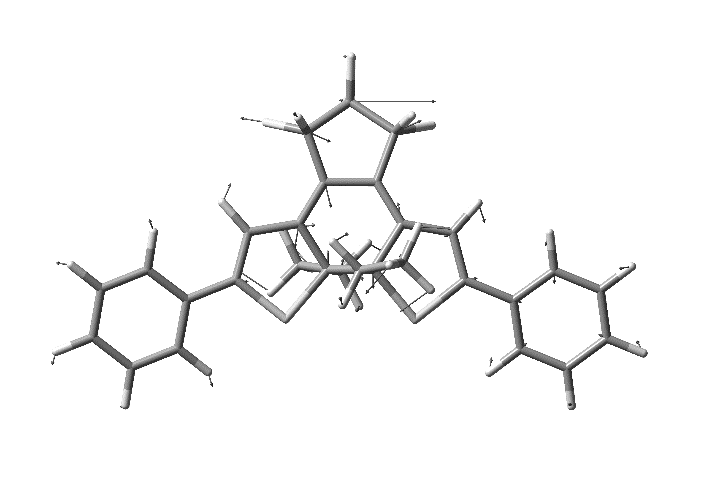 | δ(CCC)_Per_+ τ(CH)_Et_+ |
| 1079 (1072) | 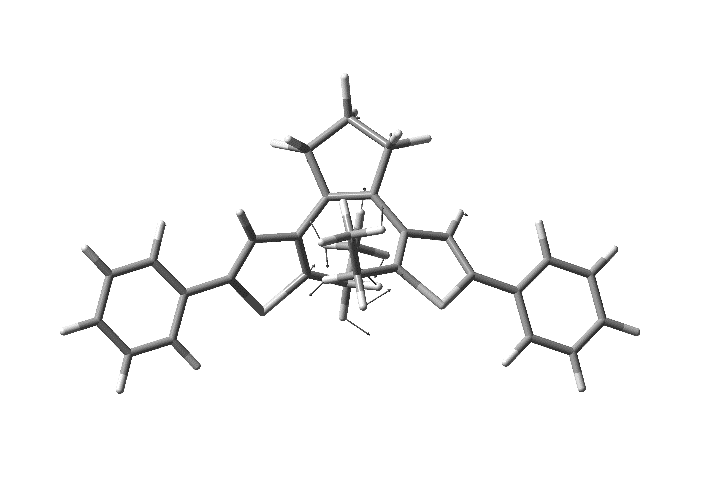 | τ(CH)_Et_ |
| 1143 (1136) | 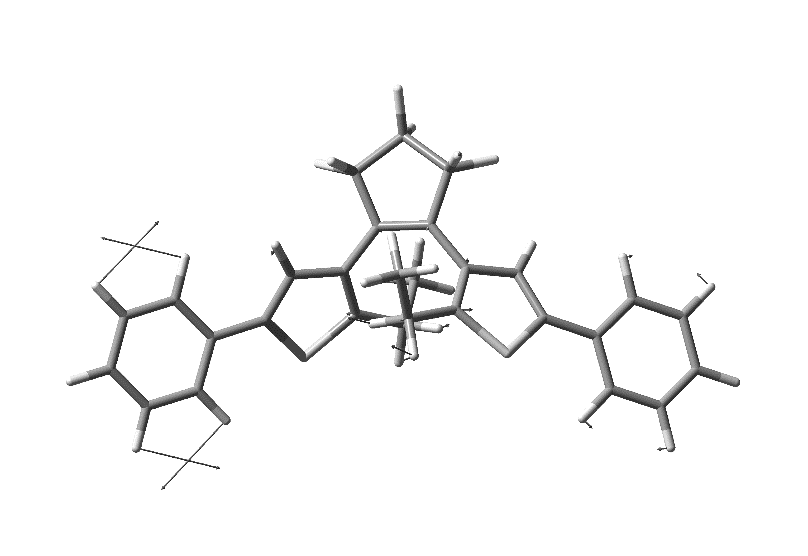 | ρ(CH)_Ph_+ ρ(CH)Thio |
| 1204 (1196) | 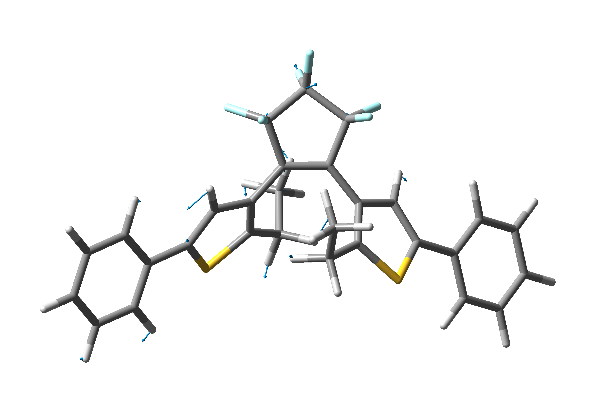 | γ(CCC)_Per_+τ(CH)_Tio_ |
| 1279 (1277) | 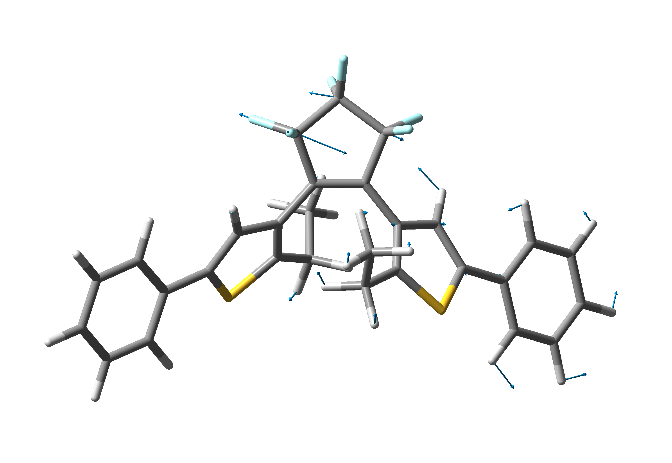 | ρ(CH)_Ph_+ ν(CC)_Per_ |
| ν: stretching, δ: in-plane bending vibration (scissoring), γ: bending out-of-plane vibration (breathing), ρ: in-plane bending (rocking), ω: out-of-plane bending (wagging), τ: out-of-plane bending (twisting) Thio: thiophene, Per: perfluorocyclopentene, Ph: phenyl, Et: ethyl | | |

| Molecule (3) closed isomer | | |
| --- | --- | --- |
| Vibrational frequency (cm^-1^) | Vibrational frequency  and its graphical representation | Assignment |
| 976 (1009) | 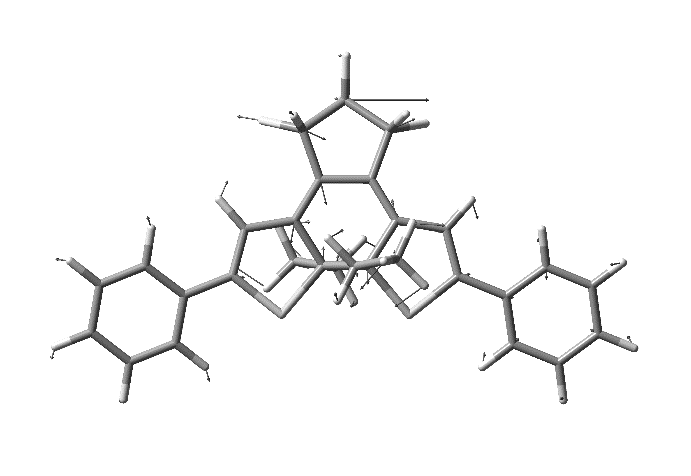 | δ(CCC)_Per_+ τ(CH)_Et_ |
| 1050 (1030) | 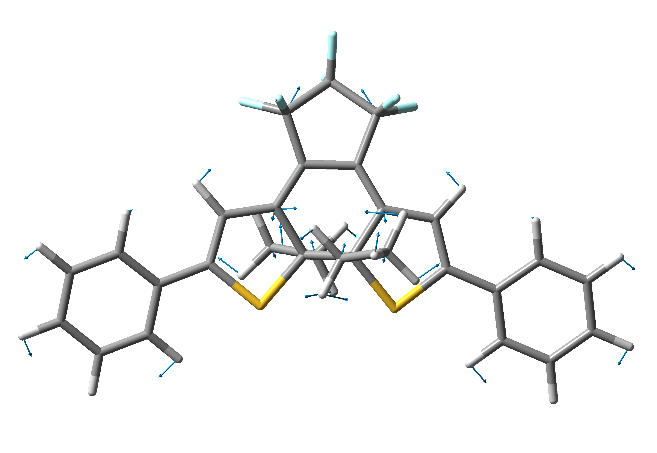 | ρ(CH)_Ph_+ ν(CC)_Per_+ τ(CH)_Et_+ω(CH)_Et_ |
| 1065 (1066) | 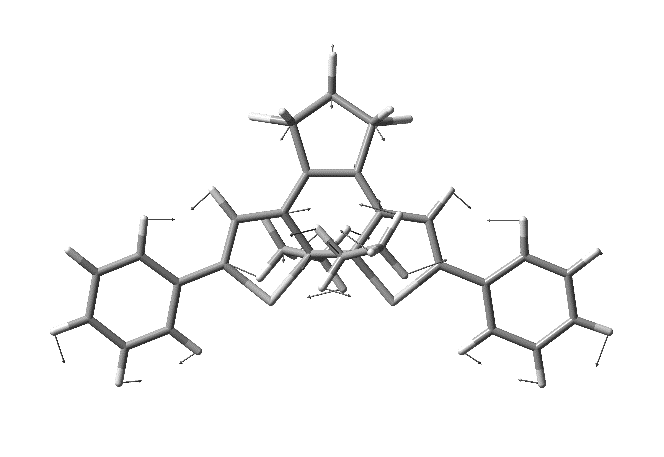 | τ(CH)_Et_+ ρ(CH)_Ph_ |
| 1145 (1112) | 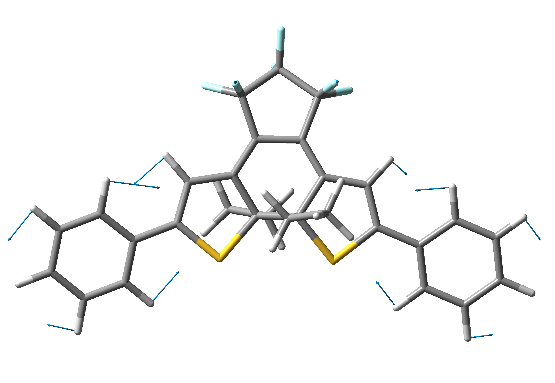 | ρ(CH)_Ph_+ ρ(CH)_Tio_ |
| 1454 (1477) | 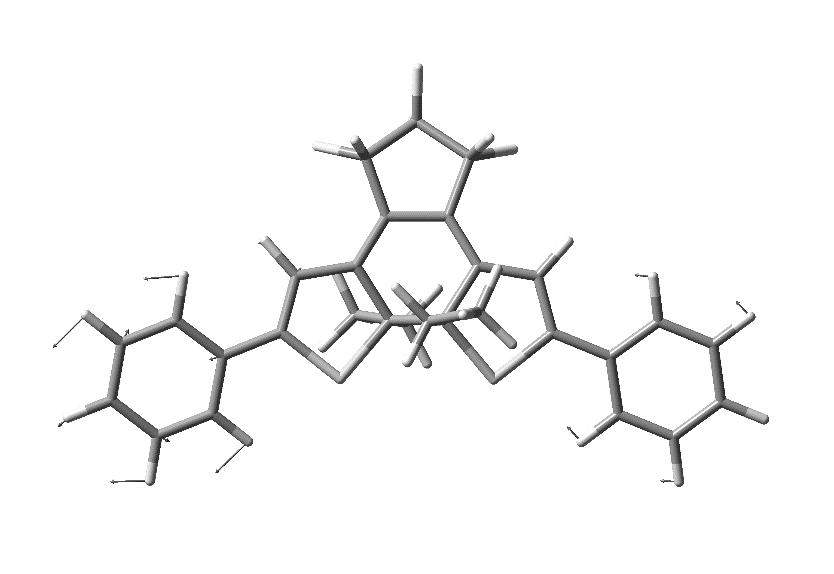 | ρ(CH)_Ph_ |
| 1513 (1500) | 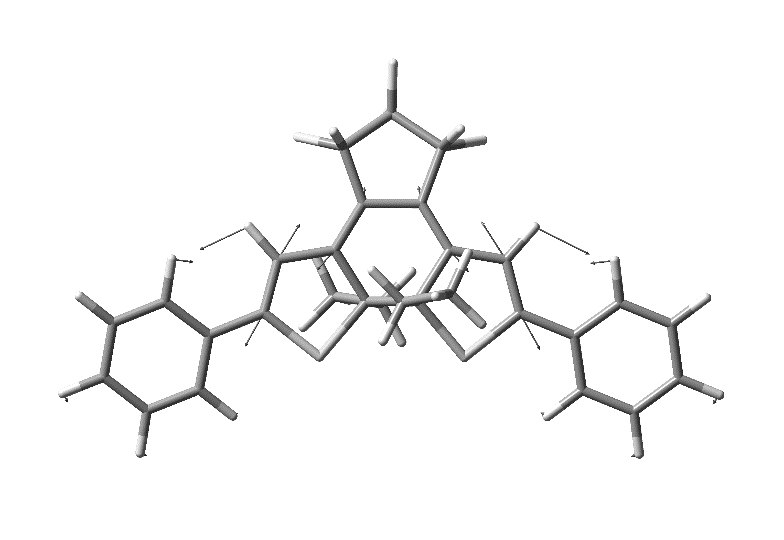 | ν(CC)_Thio_+ ν(CC)_Thio_ |
| ν: stretching, δ: in-plane bending vibration (scissoring), γ: bending out-of-plane vibration (breathing), ρ: in-plane bending (rocking), ω: out-of-plane bending (wagging), τ: out-of-plane bending (twisting) Thio: tiophen, Per: perfluorocyclopentene, Ph: phenyl, Et: ethyl, CR: central ring | | |

| Molecule (4) open isomer | | |
| --- | --- | --- |
| Vibrational frequency (cm^-1^) | Vibrational frequency  and its graphical representation | Assignment |
| 1120 (1132) | 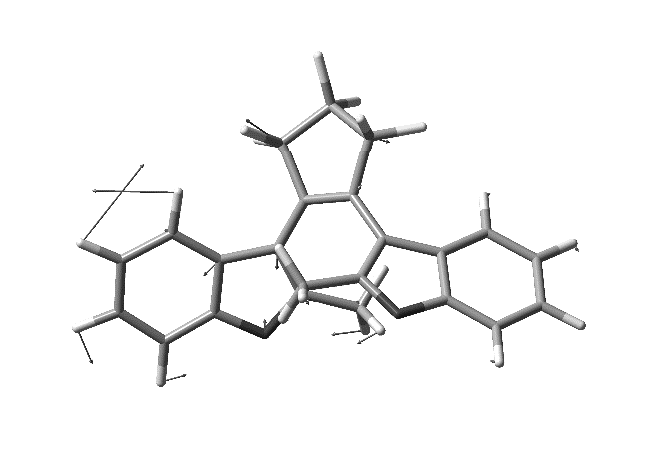 | ρ(CH)_Ph_+ ν(CC)_Per_ |
| 1183 (1195) | 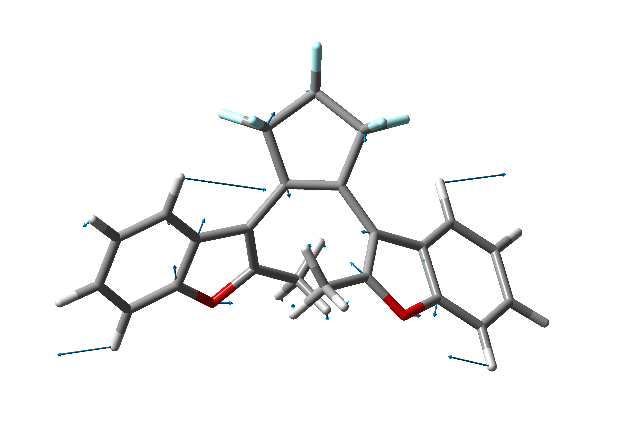 | ρ(CH)_Ph_+ ω(CH)_Fur_ |
| 1239 (1250) | 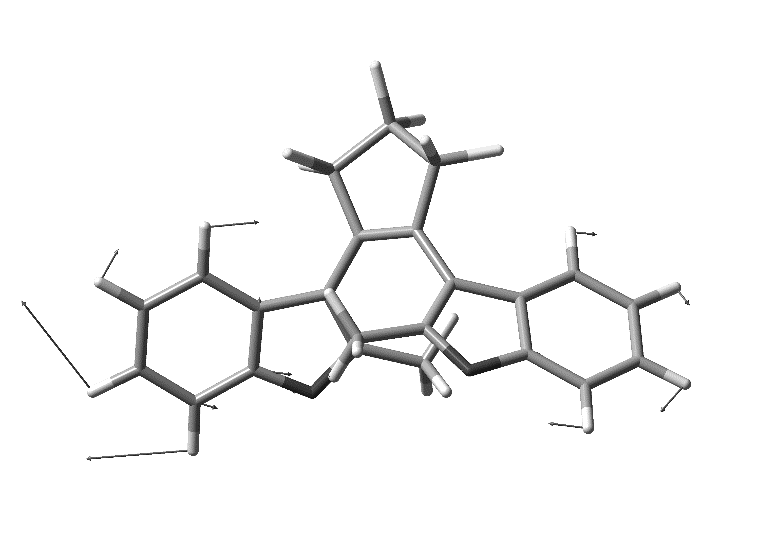 | ρ(CH)_Ph_ |
| 1279 (1279) | 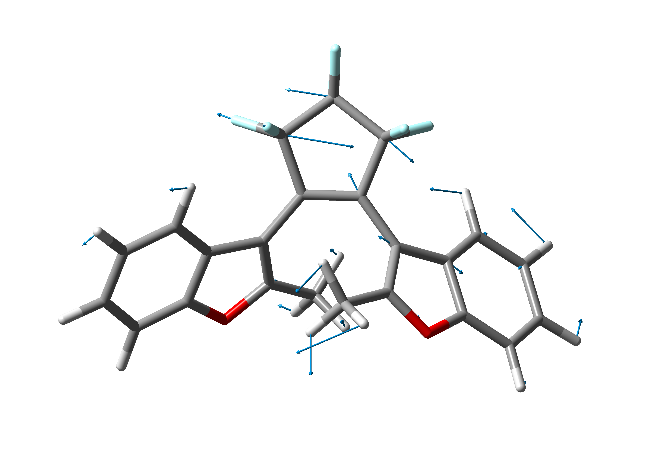 | γ_ring(Per)_+ ρ(CH)_Ph_+ γ_CH3(Ph)_ |
| 1460 (1456) | 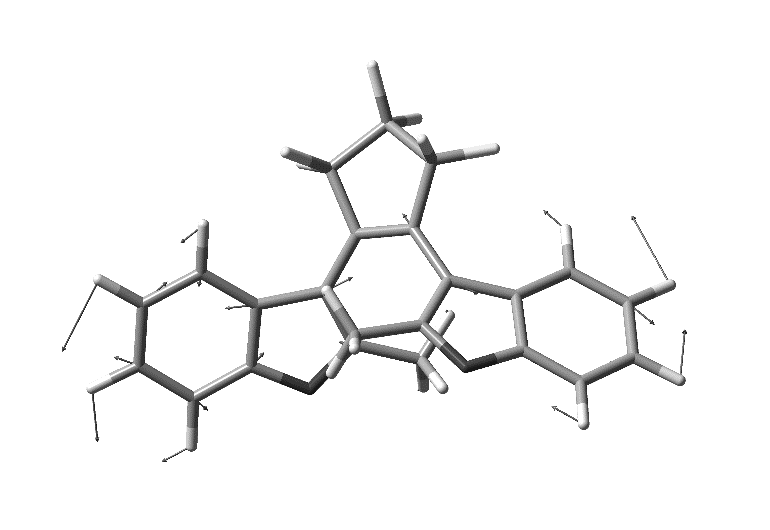 | ν(CC)_Fur_+ ν(CC)_Ph_+ ρ(CH)_Ph_ |
| ν: stretching, δ: in-plane bending vibration (scissoring), γ: bending out-of-plane vibration (breathing), ρ: in-plane bending (rocking), ω: out-of-plane bending (wagging), τ: out-of-plane bending (twisting), Per: perfluorocyclopentene, Ph: phenyl, Et: ethyl, Fur: Furan | | |

| Molecule (4) closed isomer | | |
| --- | --- | --- |
| Vibrational frequency (cm^-1^) | Vibrational frequency  and its graphical representation | Assignment |
| 974 (968) | 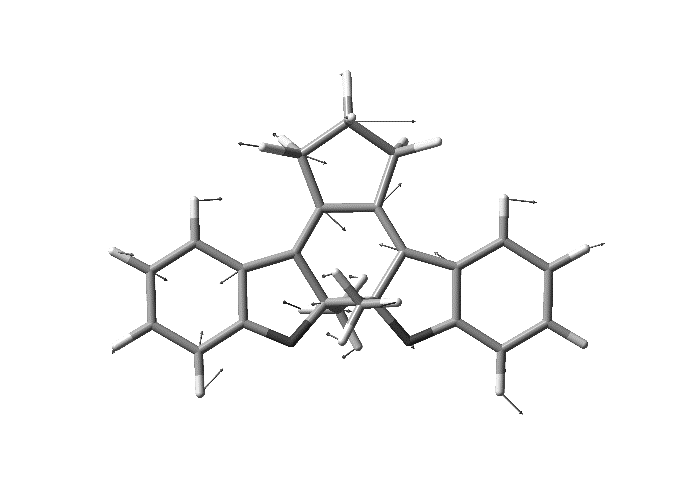 | δ(CCC)_Per_+ δ(CCC)_CR_+ ν(CC)_Ph_ |
| 1052 (1050) | 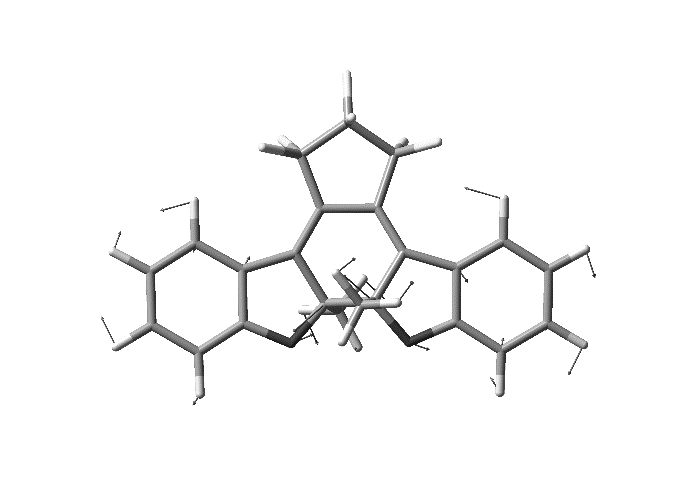 | ν(CC)_Fur_+ ν(CS)_Fur_+ ρ(CH)_Ph_ |
| 1163 (1134) | 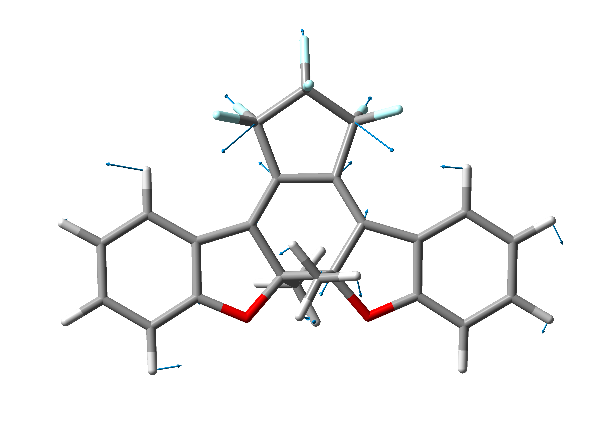 | γ_ring(Per)_+ ρ(CH)_Ph_ |
| 1210 (1192) | 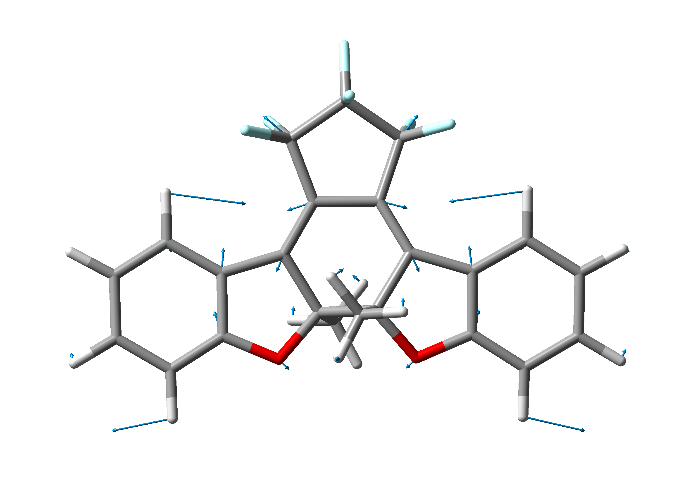 | ν(CC)_Per_+ ν(CC)_CR_ |
| 1224 (1257) | 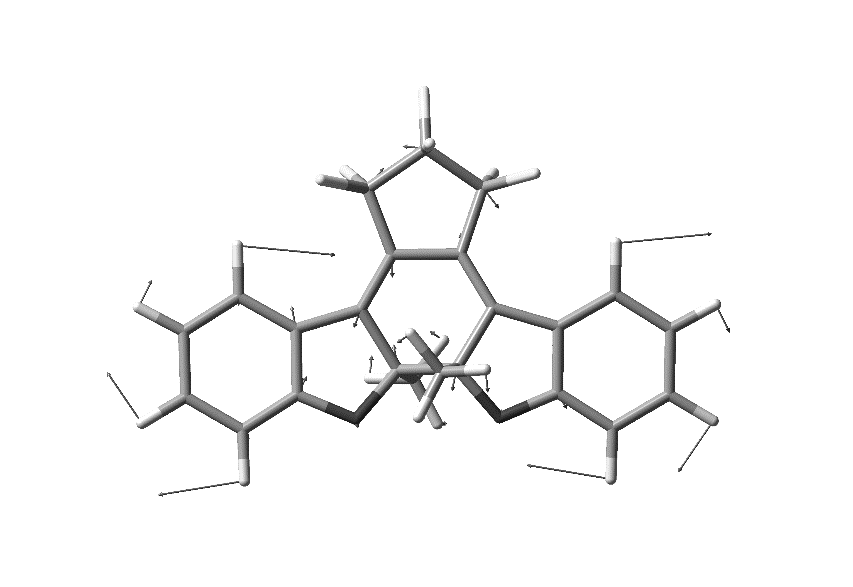 | ν(CC)_Per_+ ν(CC)_Fur_+ ρ(CH)_Ph_ |
| 1326 (1327) | 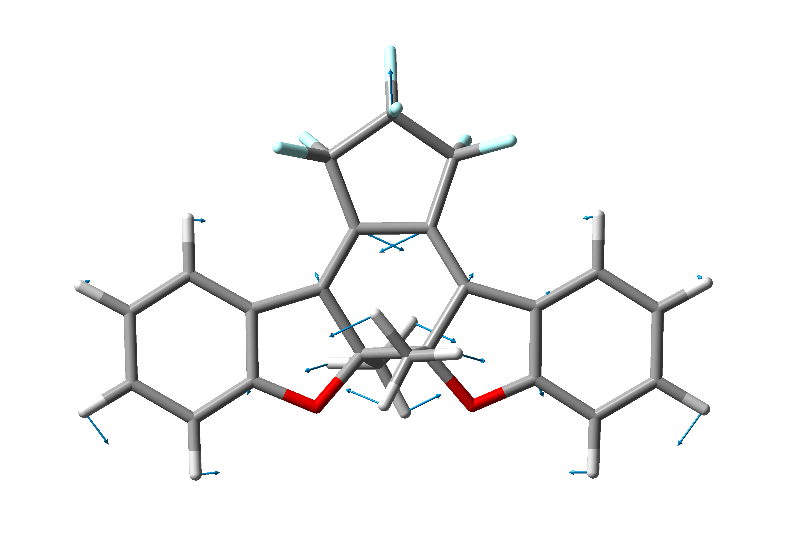 | ν(CC)_Per_+ ν(CC)_CR_+ ρ(CH)_Ph_ |
| 1610 (1607) | 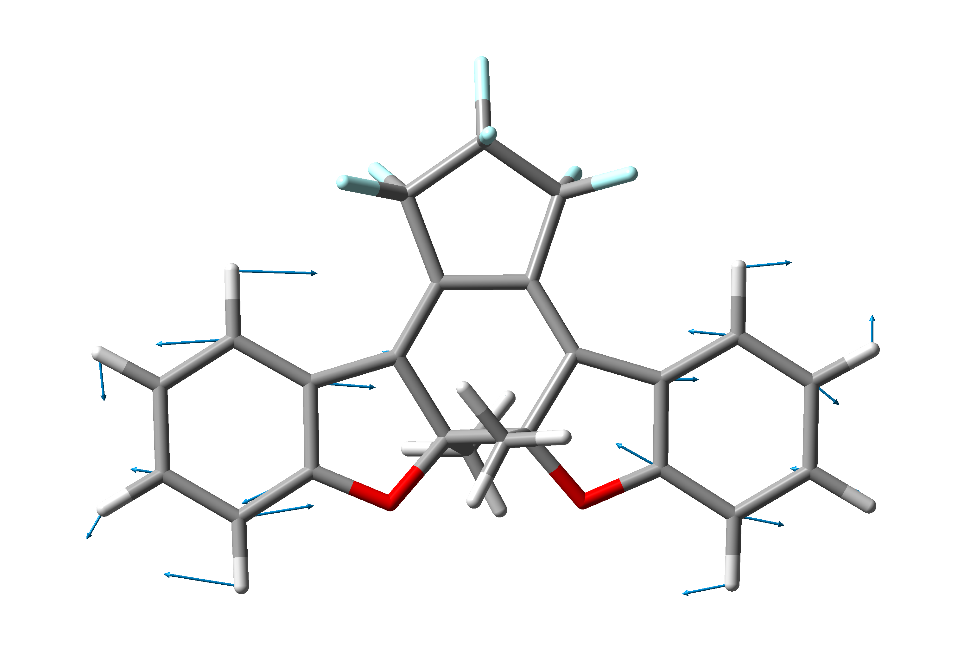 | ν(CC)_Tio_+ ν(CC)_CR_+ ρ(CH)_Ph_ |
| 1673 (1667) | 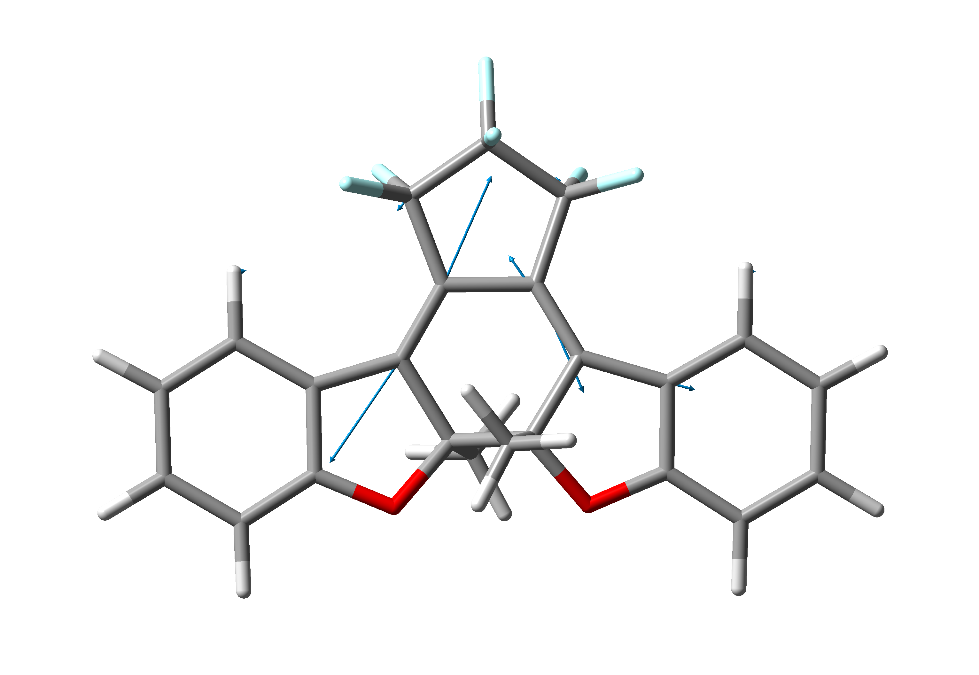 | ν(CC)_CR_+ ν(CC)_Tio_ |
| ν: stretching, δ: in-plane bending vibration (scissoring), γ: bending out-of-plane vibration (breathing), ρ: in-plane bending (rocking), ω: out-of-plane bending (wagging), τ: out-of-plane bending (twisting), Per: perfluorocyclopentene, Ph: phenyl, Et: ethyl, Fur: Furan, CR: central ring | | |

1. The results of tests of the script

Table S2. MAEs and number of found peaks for molecule 2 (open isomer) using the attached script

| Parameters set | tol=40, htol=3,  to_assign=20 | | tol=40, htol=2.5,  to_assign=20 | | tol=40, htol=2,  to_assign=20 | | tol=40, htol=3.5,  to_assign=20 | |
| --- | --- | --- | --- | --- | --- | --- | --- | --- |
|  | MAE | Number of found peaks | MAE | Number of found peaks | MAE | Number of found peaks | MAE | Number of found peaks |
| **PBE0** | 12.38 | 15 | 13.19 | 14 | 14.2 | 14 | 11.94 | 18 |
| **PBE0-D3** | 10.39 | 16 | 11.53 | 15 | 11.53 | 15 | 11.27 | 18 |
| **B3LYP** | 16.34 | 17 | 15.58 | 16 | 18.81 | 15 | 15.87 | 18 |
| **B3LYP -D3** | 14.49 | 18 | 15.03 | 17 | 16.5 | 16 | 15.1 | 19 |
| **CAM- B3LYP** | 14.28 | 18 | 14.01 | 17 | 15.59 | 15 | 12.83 | 19 |
| **CAM- B3LYP-D3** | 12.08 | 18 | 12.95 | 17 | 12.4 | 15 | 12.26 | 19 |
| **M06L** | 17.60 | 17 | 18.28 | 15 | 20.58 | 15 | 13.45 | 16 |
| **ωB97-XD** | 12.77 | 18 | 13.31 | 17 | 12.81 | 14 | 13.3 | 18 |
| **LC-ωPBE** | 10.54 | 13 | 9.95 | 12 | 9.95 | 12 | 11.56 | 15 |
| **LC-ωPBE -D3** | 12.1 | 14 | 12.1 | 14 | 12.67 | 13 | 13.4 | 15 |

Table S2a. MAEs and number of found peaks molecule 2 (open isomer) using the attached script

| Parameters set: | tol=40, htol=4,  to_assign=20 | | tol=60, htol=3,  to_assign=20 | | tol=40, htol=3,  to_assign=27 | | tol=30, htol=2,  to_assign=20 | |
| --- | --- | --- | --- | --- | --- | --- | --- | --- |
|  | MAE | Number of found peaks | MAE | Number of found peaks | MAE | Number of found peaks | MAE | Number of found peaks |
| **PBE0** | 12.26 | 18 | 20.51 | 18 | 13.63 | 19 | 13.85 | 17 |
| **PBE0-D3** | 11.27 | 18 | 16.67 | 18 | 11.17 | 20 | 10.75 | 18 |
| **B3LYP** | 16.03 | 19 | 19.82 | 18 | 17.02 | 21 | 13.8 | 14 |
| **B3LYP -D3** | 15.1 | 19 | 19.81 | 20 | 16.24 | 23 | 13.97 | 17 |
| **CAM- B3LYP** | 12.83 | 19 | 18.02 | 19 | 15.76 | 22 | 10.58 | 13 |
| **CAM- B3LYP-D3** | 12.26 | 19 | 15.88 | 19 | 13.66 | 21 | 8.96 | 14 |
| **M06L** | 13.45 | 16 | 17.97 | 17 | 17.29 | 21 | 16.25 | 14 |
| **ωB97-XD** | 13.53 | 19 | 12.77 | 18 | 13.75 | 22 | 9.74 | 15 |
| **LC-ωPBE** | 11.56 | 15 | 16.92 | 16 | 11.5 | 15 | 7.7 | 12 |
| **LC-ωPBE -D3** | 13.4 | 15 | 16.92 | 16 | 12.8 | 16 | 9.85 | 13 |

Table S2b. MAEs and numbers of found peaks molecule 2 (open isomer) using the attached script

| Parameters set: | tol=50, htol=4,  to_assign=27 | | tol=50, htol=4,  to_assign=20 | | tol=10, htol=3,  to_assign=20 | | tol=20, htol=3,  to_assign=20 | |
| --- | --- | --- | --- | --- | --- | --- | --- | --- |
|  | MAE | Number of found peaks | MAE | Number of found peaks | MAE | Number of found peaks | MAE | Number of found peaks |
| **PBE0** | 14.66 | 23 | 13.89 | 19 | 6.1 | 9 | 8.66 | 12 |
| **PBE0-D3** | 13.19 | 23 | 12.96 | 19 | 5.54 | 10 | 8.11 | 14 |
| **B3LYP** | 16.70 | 23 | 16.03 | 19 | 5.33 | 7 | 8.20 | 10 |
| **B3LYP-D3** | 16.65 | 24 | 15.10 | 19 | 5.31 | 7 | 10.19 | 14 |
| **CAM- B3LYP** | 14.8 | 24 | 12.83 | 19 | 4 | 9 | 6.40 | 12 |
| **CAM- B3LYP-D3** | 14.05 | 23 | 12.26 | 19 | 4.57 | 12 | 5.56 | 13 |
| **M06L** | 19.31 | 23 | 17.14 | 18 | 4.56 | 6 | 8.96 | 9 |
| **ωB97-XD** | 14.34 | 23 | 13.53 | 19 | 6.16 | 10 | 8.28 | 14 |
| **LC-ωPBE** | 13.88 | 18 | 13.40 | 16 | 4.51 | 9 | 6.77 | 11 |
| **LC-ωPBE-D3** | 15.91 | 18 | 13.40 | 15 | 4.41 | 9 | 6.34 | 11 |

1. Mean signed errors ( $\sum\frac{\nu_{predict}-\nu_{exp}}{N}$ ) of the simulated spectra

Table S3. MSEs for all the tested functionals

| Functional | Full spectral range (400-4000 cm^-1^) | Low spectral range (400-1000 cm^-1^) | Middle and high spectral range (1000-4000 cm^-1^) |
| --- | --- | --- | --- |
| **PBE0** | -1.94 | -8.35 | 0.54 |
| **PBE0-D3** | -2.93 | -7.83 | -1.02 |
| **B3LYP** | -6.42 | -9.88 | -5.01 |
| **B3LYP-D3** | -3.87 | -9.00 | -1.90 |
| **CAM- B3LYP** | -2.92 | -6.26 | -1.59 |
| **CAM- B3LYP-D3** | -1.83 | -4.65 | -0.69 |
| **M06L** | -5.28 | -9.64 | -3.47 |
| **ωB97-XD** | -2.06 | -7.09 | -0.10 |
| **LC-ωPBE** | -2.59 | -5.34 | -1.48 |
| **LC-ωPBE-D3** | -1.90 | -4.71 | -0.70 |
